# Membrane Topology and Biochemical Characterization of the *Escherichia coli* BacA Undecaprenyl-Pyrophosphate Phosphatase

**DOI:** 10.1371/journal.pone.0142870

**Published:** 2015-11-11

**Authors:** Guillaume Manat, Meriem El Ghachi, Rodolphe Auger, Karima Baouche, Samir Olatunji, Frédéric Kerff, Thierry Touzé, Dominique Mengin-Lecreulx, Ahmed Bouhss

**Affiliations:** 1 Institute for Integrative Biology of the Cell (I2BC), UMR 9198, CEA, CNRS, Université Paris Sud, Bâtiment 430, F-91400, Orsay, France; 2 Centre d'Ingénierie des Protéines, Université de Liège, Institut de Physique B5a et Institut de Chimie B6a, Sart-Tilman, B-4000, Liège, Belgium; Centre National de la Recherche Scientifique, Aix-Marseille Université, FRANCE

## Abstract

Several integral membrane proteins exhibiting undecaprenyl-pyrophosphate (C_55_-PP) phosphatase activity were previously identified in *Escherichia coli* that belonged to two distinct protein families: the BacA protein, which accounts for 75% of the C_55_-PP phosphatase activity detected in *E*. *coli* cell membranes, and three members of the PAP2 phosphatidic acid phosphatase family, namely PgpB, YbjG and LpxT. This dephosphorylation step is required to provide the C_55_-P carrier lipid which plays a central role in the biosynthesis of various cell wall polymers. We here report detailed investigations of the biochemical properties and membrane topology of the BacA protein. Optimal activity conditions were determined and a narrow-range substrate specificity with a clear preference for C_55_-PP was observed for this enzyme. Alignments of BacA protein sequences revealed two particularly well-conserved regions and several invariant residues whose role in enzyme activity was questioned by using a site-directed mutagenesis approach and complementary *in vitro* and *in vivo* activity assays. Three essential residues Glu21, Ser27, and Arg174 were identified, allowing us to propose a catalytic mechanism for this enzyme. The membrane topology of the BacA protein determined here experimentally did not validate previous program-based predicted models. It comprises seven transmembrane segments and contains in particular two large periplasmic loops carrying the highly-conserved active site residues. Our data thus provide evidence that all the different *E*. *coli* C_55_-PP phosphatases identified to date (BacA and PAP2) catalyze the dephosphorylation of C_55_-PP molecules on the same (outer) side of the plasma membrane.

## Introduction

**Fig 1:**
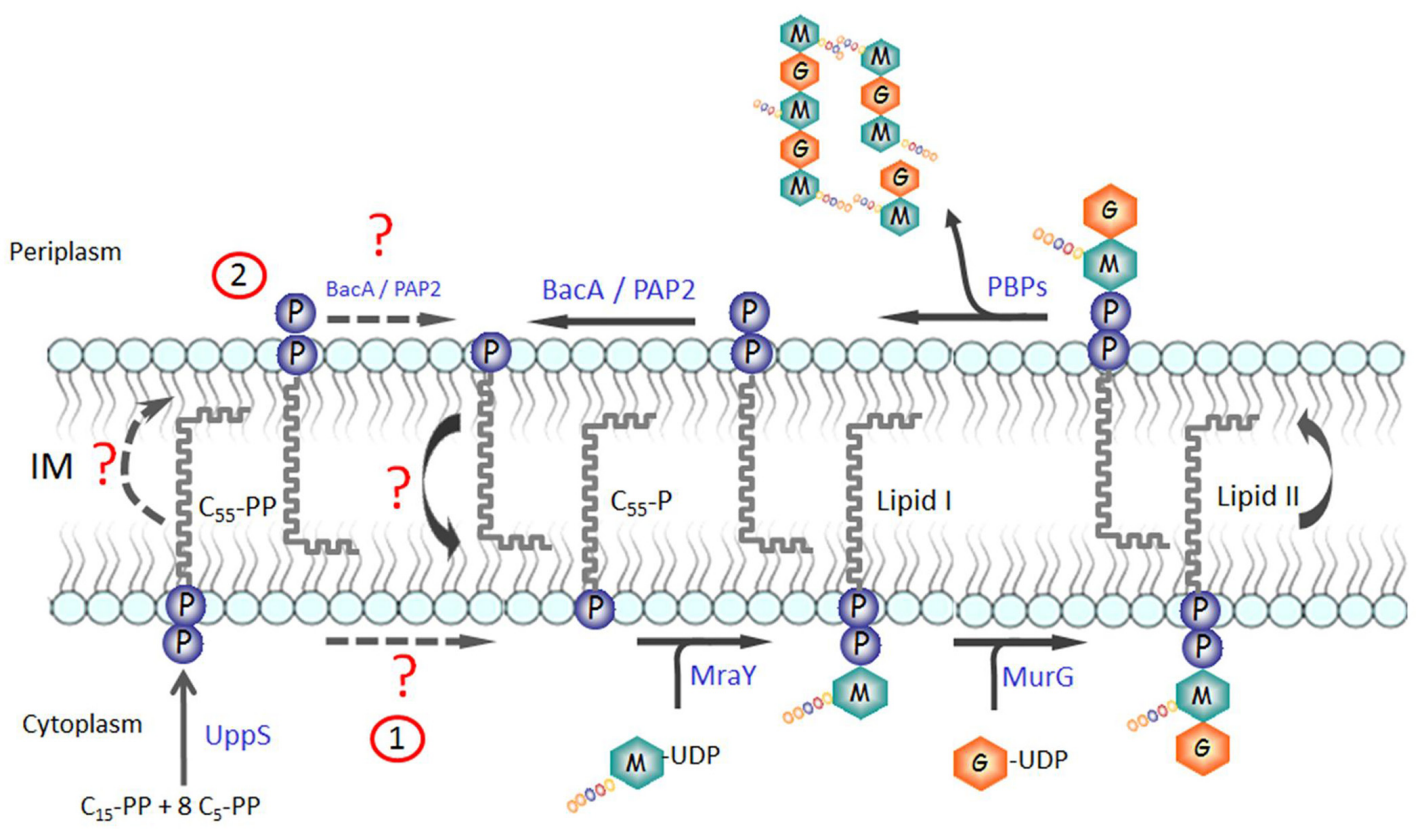
Biosynthesis, use and recycling of C_55_-P carrier lipid. The C_55_-PP precursor is *de novo* synthesized by the UppS enzyme in the cytoplasm, from which it partitions in the inner side of the plasma membrane. Then, it must be dephosphorylated to be used as a lipid carrier for the biosynthesis of various cell-envelope polymers such as the peptidoglycan, as exemplified here. As discussed in the text, two different hypotheses can be envisaged for the dephosphorylation of the *de novo*-synthesized C_55_-PP, which are illustrated here. Once the glycan has been transferred onto an acceptor in the periplasm, the lipid carrier is released again in its pyrophosphate form, which must be dephosphorylated and shuttled back to the inner side of the membrane to be reused.

Undecaprenyl-phosphate (C_55_-P) is an essential lipid present in the bacterial cell membrane that plays a central role in the biosynthesis of various cell wall components such as the peptidoglycan, teichoic acids, and the O-antigen moiety of lipopolysaccharides [1, 2]. It indeed acts as a carrier and membrane anchor for the sugar and glycan chain building blocks of these cell envelope components [1, 3]. C_55_-P-linked intermediate precursors are synthesized on the cytoplasmic side of the membrane, which are then translocated by dedicated systems (flippases) to the periplasmic side of the membrane where subsequent biosynthetic steps and polymerization reactions occur. For instance, in the peptidoglycan pathway, MraY and MurG enzymes successively transfer phospho-*N*-acetylmuramoyl-pentapeptide (MurNAc-pentapeptide) and *N*-acetylglucosamine (GlcNAc) motifs, respectively, from the corresponding UDP-linked nucleotide precursors, onto the C_55_-P lipid, thereby generating the characteristic lipid II peptidoglycan precursor consisting of C_55_-PP-MurNAc(-pentapeptide)-GlcNAc (Fig 1). This intermediate is then translocated to the membrane outer leaflet by a flippase (several putative flippases have been identified [4, 5]), where the disaccharide-pentapeptide motifs are finally polymerized and cross-linked by transglycosylase and transpeptidase activities of the penicillin-binding proteins [2]. At the end of these reactions, the carrier lipid is released in an undecaprenyl-pyrophosphate (C_55_-PP), “inactive” form which could not serve as glycan acceptor and should then be recycled to participate in new rounds of peptidoglycan (and other polymers) synthesis. This recycling process requires both a dephosphorylation step, to regenerate the C_55_-P active form, and a translocation step, to drive it back to the inner side of the membrane. The *de novo* synthesis of C_55_-P also results from the dephosphorylation of its precursor C_55_-PP, which itself is formed by the condensation of eight molecules of isopentenyl-pyrophosphate (C_5_-PP) with one molecule of farnesyl-pyrophosphate (C_15_-PP), a reaction catalyzed in the cytoplasm by the UppS synthase [6, 7] (Fig 1).

The fact that the C_55_-PP dephosphorylation reaction potentially occurs on both sides of the membrane suggested the existence of different C_55_-PP phosphatases, with either cytoplasm- or periplasm-orientated active sites, that should be specifically involved in the *de novo* synthesis or the recycling of the C_55_-P carrier lipid, respectively [1]. The *bacA* gene was the first gene identified as encoding a bacterial C_55_-PP phosphatase [8]. Its overexpression in *Escherichia coli* had been earlier shown to result in the cell resistance to bacitracin [9], an antibiotic known to tightly bind to the pyrophosphate group of C_55_-PP, thereby sequestrating and blocking the dephosphorylation of this carrier lipid precursor [10]. The BacA protein (also designated as UppP), which is an integral membrane protein, was then purified to homogeneity and its C_55_-PP phosphatase activity was demonstrated *in vitro* [8]. The successful inactivation of the *bacA* gene in the *E*. *coli* chromosome showed that this gene was not essential for growth and the detection of *ca*. 25% of residual C_55_-PP phosphatase activity in mutant cell membranes suggested that other cell proteins may exist that exhibit the same activity and could maintain the cell viability in these conditions. Several genes coding for members of the PAP2 phosphatase protein family were indeed subsequently identified in *E*. *coli*, namely *pgpB*, *ybjG* and *yeiU*, that all coded for integral membrane proteins endowed with C_55_-PP phosphatase activity [11]. None of these genes was individually essential for growth and only the deletion of the three genes *bacA*, *pgpB* and *ybjG* yielded a lethal phenotype. A thermosensitive conditional strain BWTsbacA was constructed which lacked all of these genes in the chromosome but contained a copy of *bacA* on a pMAK705-derivative plasmid that could not replicate at 42°C. This mutant lysed when grown at this non permissive temperature, a phenomena which had been clearly correlated with the inhibition of C_55_-PP recycling and the resulting arrest of peptidoglycan biosynthesis [11].

The latter studies thus revealed that multiple C_55_-PP phosphatases, belonging to two unrelated BacA and PAP2 protein families, coexisted in *E*. *coli*, and that several orthologs from both families were also encountered in most other bacterial species. This raised the question of the physiological role of this redundancy. Different hypotheses can be proposed, as discussed in a recent review [1]. First, it can be speculated that some of these phosphatases are really specific of C_55_-P metabolism and that some others have a distinct function but could accept C_55_-PP to some extent as an alternative substrate, due to a broad specificity. Also, as discovered recently for the YeiU C_55_-PP phosphatase, some of these proteins could potentially exert a dual function *in vivo*. The latter enzyme, renamed LpxT, was indeed shown to specifically transfer the phosphate group from C_55_-PP onto the lipid A moiety of lipopolysaccharides [12, 13]. That other members of this protein family could also act as phosphotransferases and be involved in different specific cell-envelope structure modifications is thus conceivable. Moreover, as mentioned above, the C_55_-PP dephosphorylation reaction is expected to occur on both sides of the membrane, *i*.*e*. in two different cell compartments, which also justify the requirement for multiple C_55_-PP phosphatase activities.

On which side of the plasma membrane and on which types of substrates are really working these enzymes *in vivo* were therefore crucial points to investigate. The membrane topology and biochemical properties of the *E*. *coli* PgpB protein were analyzed first [14]. To precisely localize the active site of this enzyme, a topological map was established by using the β-lactamase fusion procedure and residues that are essential for the enzyme activity were identified by a site-directed mutagenesis approach. PgpB was shown to be constituted of six transmembrane segments and to have its N- and C-terminal ends in the cytoplasm and the active site located on the outer side of the membrane [14]. This topology has been recently fully validated by the determination of the crystal structure of this *E*. *coli* enzyme [15]. The pure PgpB protein was shown *in vitro* to exhibit a broad-range substrate specificity as it could efficiently dephosphorylate various lipid pyrophosphate molecules, including C_55_-PP [14]. Tatar *et al*. analyzed to some extent the membrane topology of YbjG and YeiU proteins, the two other *E*. *coli* C_55_-PP phosphatases belonging to the PAP2 family, and they found that the active sites of these enzymes were orientated towards the periplasm, as it was the case for PgpB [16]. This suggested that all these PAP2 phosphatases were involved in the recycling of C_55_-PP molecules formed in the periplasmic environment. It was thus tempting to speculate that the BacA phosphatase could be responsible for the dephosphorylation of the *de novo* synthesized C_55_-PP molecules that are generated by the UppS synthase in the cytosol. Using various modeling programs, several authors previously reported some predictions of the structural organization of the BacA protein in the membrane. In their 2007 paper, Tatar *et al*. predicted that the BacA active site should be in the cytosol [16]. In 2013, Bickford and Nick went to the same conclusion and also predicted that BacA should contain seven transmembrane segments and have its C-terminal extremity in the periplasm [17]. More recently, Chang *et al*. proposed a completely different model in which the active site was in the periplasm and the protein constituted of eight transmembrane segments with both its N- and C-terminal extremities in the cytosol [18]. However, none of these models was supported by any experimental data and on which side of the membrane the BacA phosphatase exerts its activity still remained to be determined. In the present work, the membrane topology of the BacA protein was for the first time elucidated experimentally and the active site and substrate specificity of this enzyme were investigated in more detail using complementary *in vitro* and *in vivo* functional assays.

## Materials and Methods

### Chemicals

Undecaprenyl-pyrophosphate (C_55_-PP) and undecaprenyl-phosphate (C_55_-P) were provided by the Institute of Biochemistry and Biophysics of the Polish Academy of Sciences. Farnesyl-pyrophosphate (C_15_-PP) and isopentenyl-pyrophosphate (C_5_-PP) were from Sigma and [^14^C]C_5_-PP was purchased from Perkin Elmer Life Sciences. Diacylglycerol-pyrophosphate was from Avanti Polar Lipids and phosphatidylglycerol-phosphate was synthesized by using purified *E*. *coli* PgsA enzyme, essentially as described by Lu *et al*. [19]. *n*-dodecyl-β-D-maltoside (DDM) was purchased from Anatrace and isopropyl-β-D-thiogalactopyranoside (IPTG) from Eurogentec. C_55_-PP synthase UppS was purified as described before [8]. DNA ligase and restriction enzymes were obtained from New England Biolabs, and DNA purification kits were from Promega and Macherey-Nagel. Synthesis of oligonucleotides and DNA sequencing were performed by Eurofins-MWG. All other materials were reagent grade and obtained from commercial sources.

### Bacterial strains, plasmids, and growth conditions

The *E*. *coli* strains DH5α (Life Science Technologies, Inc.), BW25113 [20], and C43(DE3) (Avidis-France) were used as hosts for plasmids and for the overproduction of the BacA enzyme. The thermosensitive conditional mutant strain BWTsbacA that carries a triple chromosomal gene deletion (Δ*bacA* Δ*ybjG* Δ*pgpB*::kan) and harbors a *bacA*-expressing plasmid pMAK*bacA* (Cm^R^) whose replication is impaired at 42°C has been previously described [11]. The p*Trc*99A vector was obtained from Amersham Biosciences and the p*Trc*Bac30 derived plasmid expressing BacA with a N-terminal 6×His-tag was described previously [8]. The pNF150 plasmid carrying the *lac* promoter, a fragment of the β-galactosidase gene and the mature form of the β-lactamase gene was obtained from J.-P. Bohin [21]. 2YT rich medium was used for growing cells [22] and growth was monitored at 600 nm with a Shimadzu UV-1601 spectrophotometer. When required, ampicillin, kanamycin and chloramphenicol were added at final concentrations of 100, 50 and 25 μg.ml^-1^, respectively.

### General DNA techniques and *E*. *coli* cell transformation

PCR amplification of genes from the *E*. *coli* chromosome was performed in a Thermocycler 60 apparatus (Bio-med) using the Expand-Fidelity polymerase from Roche. DNA fragments were purified using the Wizard PCR Preps DNA purification kit (Promega). Plasmid isolations were carried out by the alkaline lysis method and standard techniques for endonuclease digestions, ligation and agarose gel electrophoresis were performed as previously described [23]. Transformation of *E*. *coli* cells with plasmid DNA was performed as described by Dagert and Ehrlich [24] or by electroporation.

### Site-directed mutagenesis

Site-directed mutagenesis of residues identified as invariant or highly conserved in the BacA protein family was performed by using the « Quikchange II XL site-directed mutagenesis kit » from Stratagene^®^ and appropriate oligonucleotides whose sequences are reported in S1 Table. These mutations were introduced directly in the p*Trc*Bac30 expression plasmid and all of them were confirmed by sequencing of the entire *bacA* gene insert.

### BacA protein membrane topology analysis

Transmembrane segments were predicted by using the TMAP program [25, 26] and a series of residues potentially localized within cytoplasmic or periplasmic hydrophilic regions were chosen as the junction sites for the construction of BacA-β-lactamase (BacA-BlaM) hybrid proteins, as follows. A series of truncated forms of *bacA* gene (at the 3’ end) were amplified from the *E*. *coli* chromosome using oligonucleotides listed in S1 Table. In all cases, the same forward primer (*bacA*-for) introduced a BamHI site 12 bp upstream from the initiation codon of *bacA* and the varying reverse primer introduced a KpnI site at the site of truncation. These PCR fragments were cleaved by BamHI and KpnI and inserted into the pNF150 plasmid vector cut by the same enzymes. In each case, the in-frame insertion led to IPTG-dependent expression of a protein consisting of the N-terminal fragment of BacA fused to the mature form (from HPETLVK to the end) of the β-lactamase via a short intermediary peptide AVPHAISSSPLR originating from the plasmid vector sequence. All of the plasmid constructs thus generated were verified by DNA sequencing. Each of them was then transformed into the DH5α strain and transformants were selected on kanamycin (pNF150 marker). The latter clones were subsequently tested for their susceptibility to ampicillin: appropriate dilutions of exponential phase cultures (*ca*. 200 cells) were plated onto 2YT plates containing or not ampicillin at 100 μg.ml^-1^ and growth was observed after 24 h of incubation at 37°C. The acquisition of ampicillin resistance was interpreted as the expression of a fusion protein with a periplasm-localized β-lactamase domain. To verify that all the types of transformants, in particular the ampicillin-sensitive ones, indeed expressed hybrid proteins, the ability of these strains to grow when patched (patch screening) on 2YT-agar containing ampicillin at 10 μg.ml^-1^ was controlled, as earlier described [27]. Membrane extracts were prepared to determine the levels of β-lactamase and C_55_-PP phosphatase activities present in these transformants. Briefly, DH5α cells carrying these different plasmids were grown exponentially at 37°C in 2YT medium (0.5 liter cultures) and harvested in the cold at an OD_600_ of *ca*. 1. They were suspended in 25 mM Tris-HCl buffer, pH 7.2, supplemented with 150 mM NaCl, 5 mM 2-mercaptoethanol and 10% glycerol (buffer A), and were disrupted with a Vibracell 72412 sonicator (Bioblock). This suspension was then centrifuged for 30 min at 200,000 × *g* with a TL100 Beckman centrifuge and the pellets of membranes were washed three times with buffer A. The extraction and solubilization of membrane proteins was achieved by treatment of this membrane extract by the *n*-dodecyl-β-D-maltopyranoside (DDM) detergent at a final concentration of 1.5%. Protein concentrations were determined with the QuantiProBCA assay kit (Sigma) and bovine serum albumin as the standard. These membrane extracts were incubated for 120 min at 4°C under agitation and the β-lactamase activity was assayed by using nitrocefin as a substrate and the spectrophotometric procedure described by Calbiochem (France Biochem, Meudon, France). Membranes extracts were incubated at 25°C in 100 mM phosphate buffer, pH 7.0, containing 50 μg.ml^-1^ nitrocefin and the absorbance at 486 nm was monitored. The molar extinction coefficient of hydrolyzed nitrocefin at 486 nm is 20,500 M^-1^.cm^-1^. One unit of activity was defined as the hydrolysis of one nanomole of nitrocefin per min.

### BacA functional complementation assay

Cells of the thermosensitive mutant strain BWTsbacA were made competent by treatment with calcium chloride and then transformed by the various plasmids to be tested. The mixture (cells and plasmid) was kept on ice for 1 h and then heated for 3 min at 42°C. 2YT medium was then added and the mixture was incubated for at least 2 h at 30°C. In each case, aliquots were plated onto two ampicillin-containing 2YT plates which were incubated at either 30°C or 42°C for 24 h. Functional complementation was judged by the capability of transformants to grow at both temperatures.

### Expression and purification of the BacA protein

C43(DE3) cells carrying the p*Trc*Bac30 plasmid (or one of its mutated derivatives) were used for the expression of the BacA protein in N-terminal His_6_-tagged form (Met-His_6_-Gly-Ser extension). Cells were grown in 2YT-ampicillin at 37°C and when the OD_600_ reached 0.7, the temperature of the culture was decreased to 22°C and protein expression was induced by addition of 1 mM IPTG. Incubation was continued overnight at this temperature and bacteria were harvested in the cold and washed with 25 mM Tris-HCl buffer, pH 7.2. Then, cells were resuspended in buffer A and were disrupted by sonication, as described above. This suspension was centrifuged for 30 min at 200,000 × *g* with a TL100 Beckman centrifuge and the pellets of membranes were washed three times with buffer A. The extraction and solubilization of membrane proteins was achieved by treatment of this membrane extract with the detergent DDM, as follows. BacA-containing membranes were resuspended in buffer A and DDM was added at a final concentration of 0.4% (8 mM). This mixture was incubated for 20 min at 4°C under agitation and then centrifuged as described above, yielding a first soluble extract (DDM1). The pellet was subjected to a second round of extraction in similar conditions except that the concentration of DDM was increased to 1.5% and that the incubation was prolonged overnight. A final centrifugation yielded a second soluble extract DDM2 which was then used for the purification of the BacA protein on nickel-nitrilotriacetate agarose (Ni^2+^-NTA, Qiagen). The extract was incubated overnight at 4°C under agitation with 2 ml of Ni-NTA polymer pre-equilibrated with 25 mM Tris-HCl buffer, pH 7.2, 300 mM NaCl, 5 mM 2-mercaptoethanol, and 20% glycerol (buffer B) supplemented with 10 mM imidazole. The mixture was then transferred into a Poly-prep chromatography column (Biorad) and the polymer was washed with buffer B containing 0.1% DDM. Elution of proteins was obtained by increasing progressively the concentration of imidazole from 10 to 300 mM. Aliquots from the different fractions were analyzed by SDS-PAGE as described previously [28]. Fractions that contained essentially the BacA protein (> 90% purity) were subjected to an additional gel-fitration purification step on Superdex 200 (HiLoad^TM^ 16/600 Superdex^TM^ 200) equilibrated in 0.1% DDM-containing buffer B. 2-ml fractions were collected and analyzed by SDS-PAGE. Pure fractions were pooled, eventually concentrated (30000 MWCO concentrator, Spin-X^R^ UF) and dialyzed (12–14 MWCO hemi-permeable membrane, Spectra/Por^R^) overnight at 4°C against 25 mM Tris-HCl buffer, pH 7.2, containing 150 mM NaCl, 2 mM 2-mercaptoethanol, 5% glycerol and 0.1% DDM (buffer C). The BacA protein concentration was estimated by measurement of the absorbance at 280 nm, based on a theoretical molar extinction coefficient of 25,565 M^-1^.cm^-1^.

Thermal stability of the BacA protein was studied by differential scanning calorimetry (DSC) on a Microcal instrument. DSC measurements were carried out with a BacA sample (0.5 mg.ml^-1^) dialyzed against 20 mM potassium phosphate buffer (pH 7.5), 200 mM NaCl, 10 mM 2-mercaptoethanol, 10% glycerol and 0.02% DDM. Buffer solution from the dialysis bath was used as a reference. Scanning was performed from 20°C to 100°C at a scan rate of 60°C.h^-1^. Baseline subtractions and data analyses were performed using Origin 7.0 software supplied by the manufacturer (Microcal).

### Synthesis of radiolabelled C_55_-PP substrate

[^14^C]C_55_-PP was synthesized using purified *E*. *coli* UppS synthase in a reaction mixture (300 μl) consisting in 100 mM Hepes buffer, pH 7.5, 100 μM C_15_-PP, 800 μM [^14^C]C_5_-PP (56 KBq), 0.5 mM MgCl_2_, 50 mM KCl, 0.1% Triton X-100, and enzyme (3 μg). The mixture was incubated at 25°C for 2 h and the completeness of the reaction was controlled by TLC analysis of an aliquot in conditions described below. [^14^C]C_55_-PP was then extracted from the reaction mixture by addition of 1 equivalent volume of 1 M pyridinium acetate, pH 4.5, and 2 volumes of butanol. It was recovered in the resulting upper phase and was subsequently dried under vacuum.

### C_55_-PP phosphatase assay

The standard assay was performed in a reaction mixture (10 μl) containing 20 mM Tris-HCl buffer, pH 7.5, 150 mM NaCl, 0.1% (2 mM) DDM, 50 μM [^14^C]C_55_-PP (900 Bq), and enzyme. When the phosphatase activity was tested at different pH, the following appropriate buffers were used: glycine-HCl (pH 3), sodium acetate (pH 4–5), bis-Tris-HCl (pH 6–7) or Tris-HCl (pH 7.5–9). In all cases, appropriate dilutions of enzyme were used so that the consumption of the substrate was less than 30%. The reaction mixture was incubated for 10 min at 37°C and the reaction was stopped by freezing in liquid nitrogen. The radiolabelled substrate (C_55_-PP) and reaction product (C_55_-P) were separated by TLC on pre-coated plates of silica gel 60 (Merck) using 2,6-dimethyl-*n*-heptanone/acetic acid/water (8:5:1, v/v/v) as a mobile phase (R*f* values of C_55_-PP and C_55_-P were 0.36 and 0.5, respectively). The radioactive spots were located and quantified with a radioactivity scanner (Rita Star, Raytest Isotopenmeβgeräte GmbH, Staubenhardt, Germany). The activity of BacA towards other (non-radiolabelled) substrates was determined by measuring the amounts of inorganic phosphate released during the reaction. The reaction mixture was as described above, in a final volume of 100 μl. After 10 min of reaction at 37°C, 0.9 ml of Malachite green (Biomol Green^TM^, Enzo Life Sciences) was added and the released phosphate was quantified by measurement of the absorbance at 620 nm. All the above assays were performed at least in triplicate and deviations were < 10% in all cases. Data were fitted to the equation *v* = *V*_max_ *S*/(*K*_m_ + *S*) by the Levenberg-Marquardt method [29], where *v* is the initial velocity and *S* the substrate concentration and values ± standard deviations at 95% of confidence were calculated.

## Results

### Biochemical characterization of the purified *E*. *coli* BacA protein

**Fig 2:**
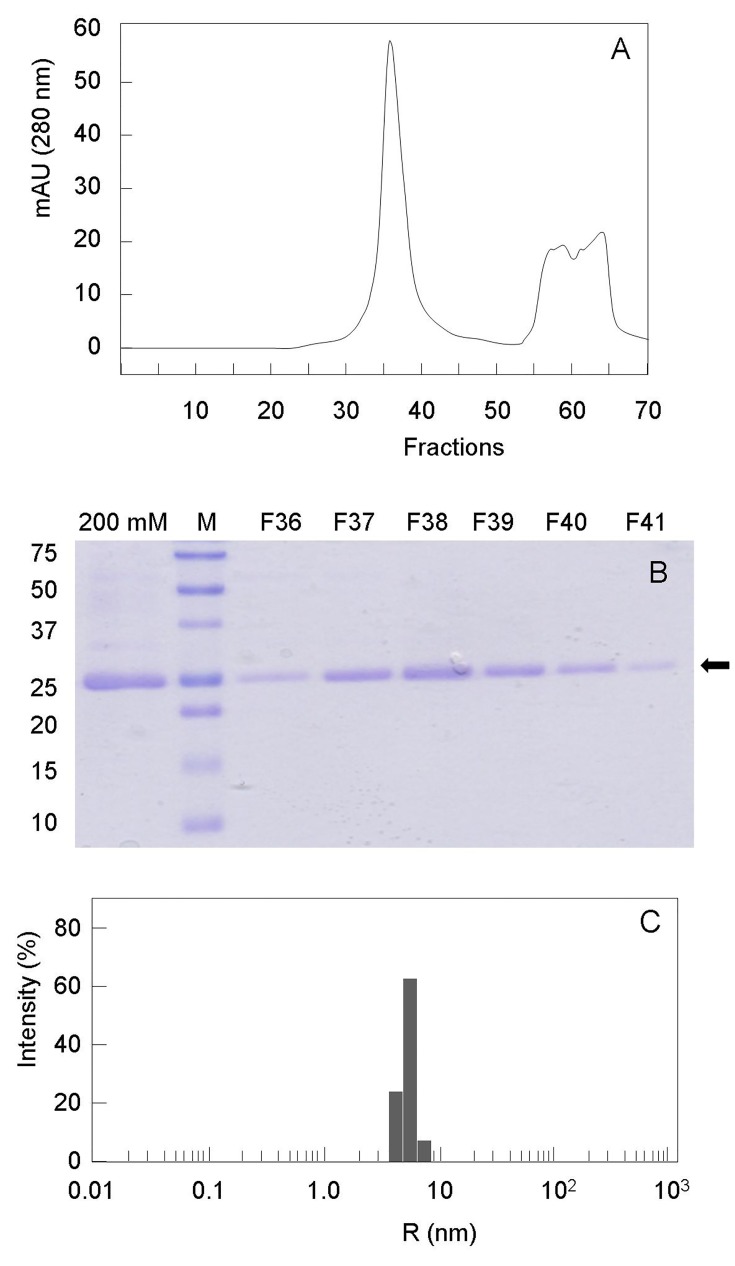
Purity and homogeneity of the BacA preparation. The *E*. *coli* BacA protein (N-terminal His-tagged form) was overproduced, extracted from cells and purified on Ni^2+^-NTA agarose as described earlier [8]. This purified material (200 mM imidazole-containing fraction) still contained some minor contaminant proteins, as judged by SDS-PAGE (panel B, first lane). It was further purified by gel filtration on a 120-ml HiLoad 16/600 Superdex 200 column, as described in the Materials and Methods (panel A). A single peak was observed, at an elution volume of 74.5 ml (main fraction: F38), corresponding to the BacA protein. It migrated with an apparent molecular mass of *ca*. 25 kDa on SDS-PAGE (12% gel, panel B), as observed previously [8]. M, molecular mass standards used are indicated on the left (kilodaltons). Panel C, Size-distribution histogram of the purified BacA protein (fraction F38) deduced from DLS experiments, showing a monodisperse size distribution.

The *E*. *coli* BacA membrane protein (His-tagged form) was overproduced to high-levels in C43(DE3) cells following IPTG-induced gene expression at low temperature (22°C) from the p*Trc*Bac30 plasmid. Previously established conditions allowing its efficient extraction and solubilization from cell membranes by the DDM detergent were used [8]. The BacA protein was then successfully purified to homogeneity by performing two successive affinity chromatography and gel-filtration steps (Fig 2). The overall production and purification yield was very good for such an integral membrane protein, *ca*. 3 mg of protein per liter of culture. This final preparation also appeared homogeneous and devoid of aggregates, as judged by dynamic light scattering (DLS) analysis (Fig 2C).

**Fig 3:**
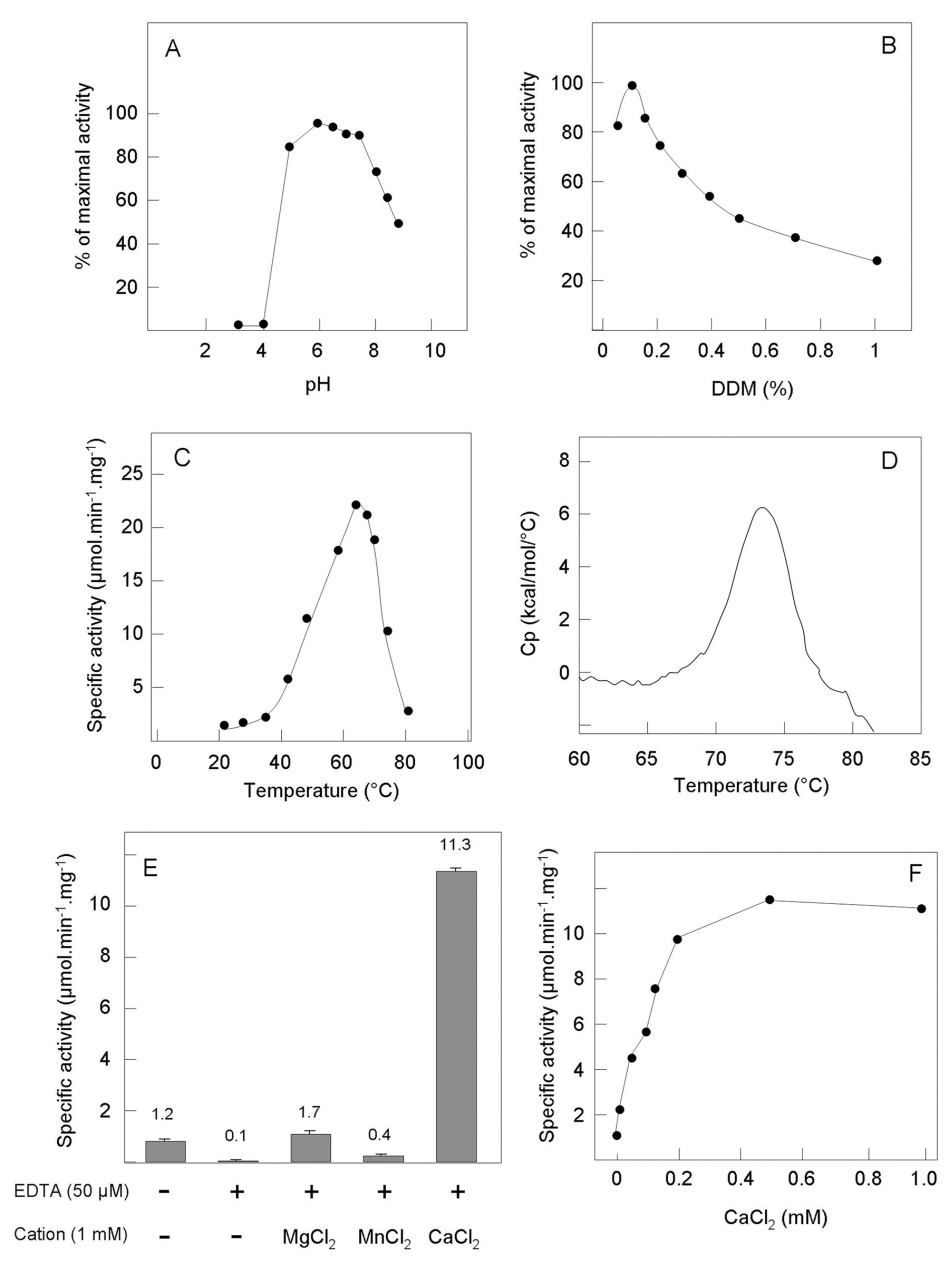
Biochemical properties of BacA C_55_-PP phosphatase activity. The effects on the BacA phosphatase activity of varying different parameters were analyzed: A, pH [pH 3 (glycine buffer), pH 4 and 5 (sodium acetate buffer), pH 6–7 (bis-Tris-HCl buffer), pH 7.5–9 (Tris-HCl buffer)]; B, DDM concentration; C, temperature. Panel D shows a typical DSC thermogram of the BacA protein (0.5 mg.ml^-1^) which displays a midpoint transition T_m_ of 73.5°C. Panel E shows the metal ion requirement of the BacA activity. The enzyme was treated or not with EDTA (50 μM) and then assayed in the presence of different divalent cations (Mg^2+^, Mn^2+^ or Ca^2+^) at a concentration of 1 mM. Panel F shows the effect on the BacA activity of varying the Ca^2+^ concentration (0–1 mM range).

The optimal assay conditions, kinetic properties and substrate specificity of the purified C_55_-PP phosphatase were further investigated, using a previously developed assay that monitors the dephosphorylation of radiolabelled [^14^C]C_55_-PP by TLC analysis [8, 11]. The specific activity of our pure enzyme preparation that we initially determined in standard assay conditions was 1.3 μmol.min^-1^.mg of protein^-1^. Then, the effects of different parameters (pH, detergent concentration, temperature, and cations) on the enzyme activity were tested. Some of these enzyme properties were also recently analyzed by Chang *et al*. using C_15_-PP, a soluble C_55_-PP analogue, as the substrate [18]. The effect of pH was determined in the 3–9 range using different buffers: BacA did not show significant activity at acid pH values < 5 and a plateau of maximal activity was observed between 5 and 7.5 (Fig 3A). Chang *et al*. also previously showed that the optimal pH of this protein was between 6.5 and 7. As a detergent is required for the solubilization of both the enzyme and the C_55_-PP substrate, the effect of varying the detergent concentration was also analyzed. The BacA activity appeared optimal at low DDM concentrations around 0.05–0.15% (1–3 mM), and it progressively decreased at higher concentrations (by *ca*. 50% at 10 mM), an effect likely due to the dilution of the C_55_-PP lipid substrate molecules in the detergent micelles rather than to an inhibition of the enzyme activity by this detergent (Fig 3B). To verify the latter assumption, we also tested the effect of another non-ionic detergent, lauryldimethylamine-oxide (LDAO), on the BacA activity towards C_55_-PP. A similar optimal activity was detected at 0.1% LDAO (4.3 mM) and the activity decreased progressively with the detergent concentration, as observed with DDM (data not shown). Moreover, when the C_15_-PP water-soluble substrate was used instead by Chang *et al*. [18], no effect of the detergent concentration was observed, thus also supporting the surface dilution effect of detergents when using a lipid substrate. The BacA activity was then tested at various temperatures ranging between 25 and 80°C. Quite interestingly, this protein appeared particularly thermostable, exhibiting an optimal activity at 65–70°C which was about 16 fold higher than at 37°C (Fig 3C). Differential scanning calorimetry (DSC) analyses confirmed that this protein was very stable, with a T_m_ of 73.5 (Fig 3D). As phosphatases sometimes require cations as cofactors, the effects of cations and EDTA on BacA activity were analyzed. Omitting MgCl_2_ in the standard reaction mixture did not affect the activity, suggesting that BacA does not require a (this) cation. However, the addition of 50 μM EDTA strongly inhibited the enzyme activity (95% inhibition) and at 1 mM of this chelator, the activity was further diminished but not totally abolished (2% residual activity) (Fig 3). This suggested that BacA required a cation for functioning and that this cation was already present in the purified enzyme stock. As no cations had been added to the buffers used for BacA purification, these cations, which remain to be identified, likely tightly bound to the protein when it was expressed in the cytosol and did not leave this position afterwards. Various cations (Mg^2+^, Mn^2+^, Zn^2+^, Co^2+^, Fe^2+^, Cu^2+^, and Ca^2+^) were then tested for their capability to restore the activity of the EDTA-pretreated enzyme. As shown in Fig 3E, the BacA activity lost after treatment with 50 μM EDTA was fully recovered following addition of MgCl_2_ and was only partially restored by MnCl_2_ (1 mM). CoCl_2_, FeCl_2_, ZnCl_2_, and CuCl_2_ did not yield significant activity. Surprisingly, in the same conditions CaCl_2_ not only restored the enzyme activity but also greatly enhanced it, by a *ca*. 9-fold factor. The latter effect was already maximal at 200 μM CaCl_2_ (Fig 3F). Chang *et al*. also recently reported a stimulating effect of Mg^2+^ and Ca^2+^ on the BacA activity using a C_15_-PP soluble substrate [18]. No significant change of the BacA protein stability was observed following the addition of either EDTA or CaCl_2_, as judged by DSC analysis. In the standard assay conditions, typical Michaelis-Menten kinetics were observed and the *K*_m_ for the physiological C_55_-PP substrate was estimated at 680 μM and the *V*_max_ at 53 μmol.min^-1^.mg of protein^-1^ (*k*_cat_ of about 27 s^-1^). The substrate specificity of this enzyme was also analyzed. BacA dephosphorylated both C_55_-PP and C_15_-PP substrates with similar efficiencies, when tested at the standard concentration of 50 μM (Table 1). Diacyl(C_8_)glycerol pyrophosphate also appeared to be a relatively good substrate. Isopentenyl-pyrophosphate (C_5_-PP), phosphatidylglycerol-phosphate and phosphatidic acid were not accepted at all (Table 1). No subsequent dephosphorylation of C_55_-P or C_15_-P products was observed (*i*.*e*. no spot corresponding to radiolabelled [^14^C]C_55_-OH was detectable by TLC), confirming that the BacA enzyme was specific for pyrophosphate substrates. This enzyme thus appeared more specific than the PgpB phosphatase which was shown to hydrolyze all of the different substrates we have tested here [14]. A *K*_m_ of 10.8 μM and a *k*_cat_ of 2.1 s^-1^ were recently determined by Chang *et al*. for BacA when using the soluble C_15_-PP substrate [18]. The concentration of 50 μM we used for this substrate was thus saturating for the enzyme. As mentioned above, when the natural C_55_-PP substrate was used instead, a 10-fold higher *k*_cat_ value (27 s^-1^) was determined, showing that the BacA phosphatase exhibited a clear preference for C_55_-PP.

**Table 1:** Substrate specificity of the purified BacA protein. The BacA activity was tested as described in Materials and Methods in the presence of 1 mM CaCl_2_, using the listed compounds at a 50 μM concentration. Results are expressed as mean ± standard deviation of three individual experiments. ND, no detectable activity, when up to 2 μg of pure BacA protein was used per assay.

Compound	BacA phosphatase activity (μmol.min^-1^.mg^-1^)
C_55_-PP	11.3 ± 1.3
C_15_-PP	10.3 ± 0.9
C_5_-PP	ND
Diacyl(C_8_)glycerol-PP	5.5 ± 0.4
Phosphatidylglycerol-P	ND
Phosphatidic acid	ND

### Characterization of BacA active site by site-directed mutagenesis

**Fig 4:**
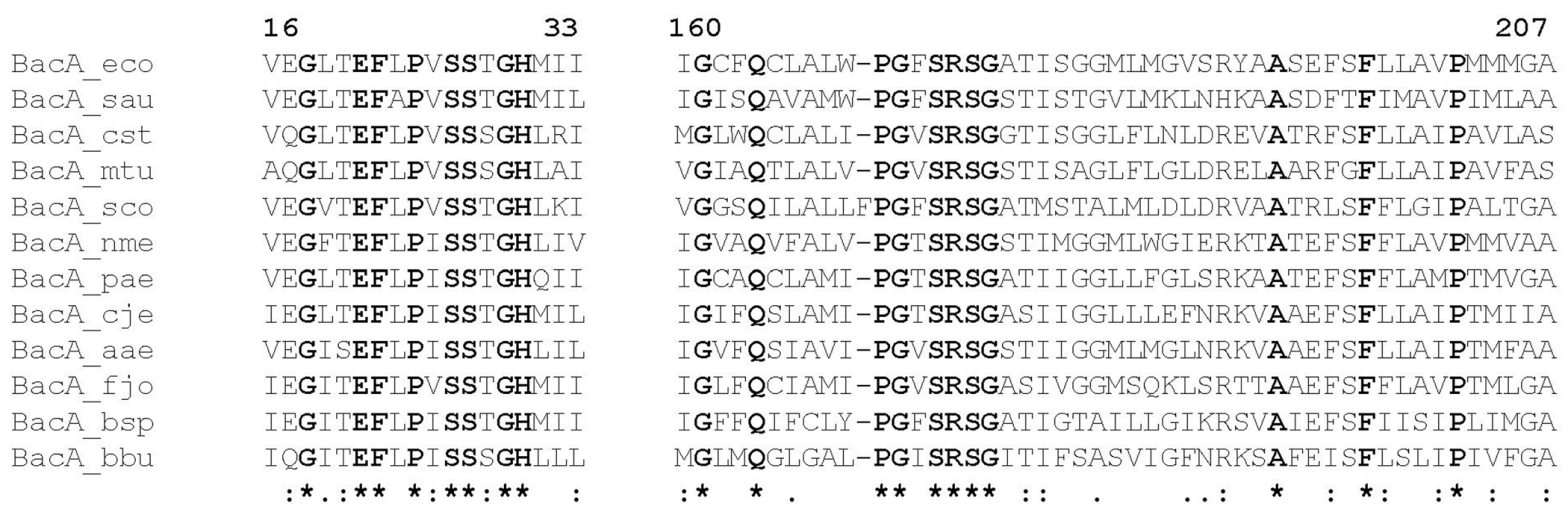
Two characteristic signature motifs detected in the BacA protein family. Alignments of these conserved motif sequences are shown for BacA orthologues from the following bacterial species: *E*. *coli* (eco), *Staphylococcus aureus* (sau), *Corynebacterium striatum* (cst), *Mycobacterium tuberculosis* (mtu), *Streptomyces coelicolor* A3 (sco), *Neisseria meningitidis* (nme), *Pseudomonas aeruginosa* (psaer), *Campylobacter jejuni* (cje), *Aquifex aeolicus* (aae), *Flavobacterium johnsoniae* (fjo), *Buchnera sp*. APS (bsp), *Borrelia burgdorferi* (bbu). Numbers indicate the position of residues in the *E*. *coli* protein. * indicates positions of single, fully-conserved residues, : indicates conservation between groups of strongly similar properties, and . indicates conservation between groups of weakly similar properties.

Contrary to the YbjG, YeiU and PgpB proteins which were classified as phosphatases since a long time, based on their homology with other members of the PAP2 phosphatase family, no putative function had been assigned to BacA until we demonstrated its C_55_-PP phosphatase activity [8]. Indeed, no characteristic signature was detected within the sequence of this protein that had been already observed in other proteins, although Bickford and Nick recently predicted that a conserved motif of BacA resembled the tyrosine phosphate phosphatase motif of the tumor suppressor, phosphatase and tensin homologue (PTEN) [17]. BacA therefore constitutes its own family of phosphatases, which is specific to the bacterial world. As previously reported by other authors [17, 18], alignments of the sequences of BacA orthologues from various bacterial species highlighted two particularly well-conserved regions Val16-Ile33 and Ile160-Ala207 that we will designate here as the BacA1 and BacA2 motifs, respectively (Fig 4).

Several invariant residues were clustered within these motifs that could potentially be involved in substrate binding and/or catalysis. The specific role of these residues, in particular those possessing a polar side-chain, was investigated by using a site-directed mutagenesis approach. The Glu21, Ser26, Ser27 and His30 residues from the BacA1 motif, and the Gln164, Ser173, Arg174, Ser175, Arg189 and Ser196 residues from the BacA2 motif, were all mutated in alanine. Site-directed mutagenesis was performed directly on the expression plasmid p*Trc*Bac30 and all the mutant proteins were produced and purified to homogeneity exactly as described for the wild-type protein, with quite similar purification yields, *i*.*e*. 2–4 mg of purified protein per liter of culture. The activity of these different BacA mutant proteins was then tested using both *in vitro* (enzymatic assay) and *in vivo* (functional complementation) assays. The specific enzyme activities of these proteins determined *in vitro* using the standard C_55_-PP phosphatase assay (Table 2) showed that with the exception of the Q164A, S175A and S196A mutants, which were only moderately affected (25–40% of the wild-type activity), all these mutations yielded dramatic losses of activity. This was particularly true for the four mutants E21A, S27A, S173A and R174A, which displayed only 1.4%, 0.013%, 2.2% and 0.13% of the wild-type activity, respectively. The mutations of Ser26, His30 and Arg189 residues also led to significant but less dramatic reductions of enzyme activity (6–15% of residual activity; Table 2). Chang *et al*. also analyzed recently the role of some of these conserved residues using a similar site-directed mutagenesis approach. The data they got are summarized in Table 2.

**Table 2:** Site-directed mutagenesis of BacA invariant residues.

BacA protein	Specific activity, this study (μmol.min^-1^.mg^-1^)^a^	Relative enzyme activity, this study (%)^b^	Functional complementation^c^	Relative enzyme activity, from Chang *et al*.[18] (%)^b^
Wild-type	10.58 ± 2.6	100	+	100
E21A	0.15 ± 0.03	1.4	-	40
S26A	1.58 ± 0.6	15	+	ND
S27A	0.0014 ± 0.0002	0.013	-	ND
H30A	0.65 ± 0.1	6.2	+	<1
Q164A	2.57 ± 0.7	24.3	+	ND
S173A	0.23 ± 0.02	2.2	+	<1
R174A	0.014 ± 0.005	0.13	-	<1
S175A	4.34 ± 0.2	41	+	32
R189A	1.28 ± 0.4	12.1	+	11
S196A	3.89 ± 0.9	36.8	+	ND

^a^ The C_55_-PP phosphatase assay was performed as described in Materials and Methods in the presence of 1 mM CaCl_2_. Results are expressed as mean ± standard deviation of three individual experiments.

^b^ Relative activities of BacA mutant proteins as compared to the wild-type protein. It should be noted that enzyme assays performed in our work and by Chang *et al*. used different substrates: C_55_-PP and C_15_-PP, respectively. ND, not done.

^c^ Plasmids expressing the wild-type or mutated *bacA* genes were tested for their capability to complement (*i*.*e*. to restore growth at 42°C of) the thermosensitive conditional mutant BWTsbacA. +, complementation; -, no complementation. In the case of the E21A, S27A and R174 mutants, the same results were observed when the gene expression was further increased by addition of IPTG (0.05 or 0.5 mM).

These different plasmids expressing wild-type or mutated copies of *bacA* were then tested for their capability to complement the BWTsbacA thermosensitive mutant strain. The three plasmids expressing the E21A, S27A and R174A mutant proteins could not complement this strain, *i*.*e*. did not restore its growth at 42°C, and the same was observed when IPTG inducer (0.05 or 0.5 mM) was added to further stimulate *bacA* gene expression from these constructs. All of the other plasmids fully complemented the thermosensitive mutant with or without IPTG. Interestingly, a very good correlation was observed between the *in vivo* (functional complementation) and *in vitro* (enzymatic activity) data. As shown in Table 2, plasmids that complemented the BWTsbacA mutant strain were those expressing a BacA mutant protein exhibiting at least 2% of the activity of the wild-type protein. All these data thus clearly showed that the His30 residue does not play a major role in the BacA reaction mechanism, contrary to what was earlier hypothesized [18]. Our work instead highlighted the crucial implication of the Ser27 residue in the catalytic process, an invariant residue which had not been selected by Chang *et al*. in the course of their site-directed mutagenesis study [18]. Another discrepancy observed between the two studies is that the latter authors reported only a minor effect for the E21A mutation (40% residual activity) while we observed it dramatically abolished the BacA activity both *in vitro* (1.4% residual activity) and *in vivo* (no complementation of the BWTsbacA mutant) (Table 2). Similar results were observed for the other mutations generated in common in the two studies (S173A, R174A, S175A and R189A) (Table 2). Then, to analyze whether the nature of the substrate could eventually explain some of the differences observed between the two studies, we also tested the activity of our BacA mutant proteins on the soluble C_15_-PP substrate. The S27A mutant did not exhibit detectable activity on this substrate. The H30A mutant appeared as active on C_15_-PP that it was on the C_55_-PP natural substrate, displaying *ca*. 6% of residual activity as compared to the wild type protein, a level higher than that reported by Chang *et al*. for this mutant (Table 2). Interestingly, we found that the E21A mutant was 20-fold more active on C_15_-PP than on C_55_-PP, which is in agreement with data from Chang *et al*. and suggests that this particular mutation also impacts in some way the enzyme substrate specificity.

### Analysis of BacA protein membrane topology

As all the C_55_-PP phosphatases from the PAP2 family identified to date were shown to have a periplasm-orientated active site, on which side of the membrane is active BacA, which belongs to a distinct protein family, was thus a crucial question. As mentioned in the Introduction, several authors previously reported some predictions of the topology of this protein but their models differed completely and none of them was validated experimentally [17, 18]. We thus decided to determine the BacA topology experimentally by using the classical procedure we earlier used successfully for MraY and PgpB integral membrane proteins [14, 30]. It consisted in the construction of a series of BacA-BlaM hybrid proteins, *i*.*e*. C-terminally truncated forms of BacA of different sizes fused to the mature form of the β-lactamase, the latter protein being used as a reporter of the BacA transmembrane topology. Indeed, if the β-lactamase is fused to a point in BacA which is in a periplasmic environment, it will be able to hydrolyze ampicillin before this antibiotic can reach its target (PBP), thereby conferring ampicillin resistance to cells. However, if it is fused to a point in BacA which is in the cytoplasm, the β-lactamase domain could no more exert its protective effect and cells will remain ampicillin sensitive.

A series of 13 BacA residues (Gly42, Gly88, His110, Lys140, Glu143, Pro144, Gly176, Ala191, Glu194, Ser215, Thr244, Arg251, and the terminal Phe273 residue) potentially localized within cytoplasmic or periplasmic hydrophilic regions based on the topology models were chosen as the junction sites for the construction of the BacA-BlaM hybrid proteins. The corresponding truncated *bacA* genes were amplified by PCR using the oligonucleotides shown in S1 Table and these fragments were inserted in the pNF150 vector as a fusion with the gene encoding the mature form of β-lactamase, as described in Materials and Methods. DH5α cells carrying these different plasmids were then tested for their susceptibility to ampicillin (100 μg.ml^-1^), when plated at a low cell density (*ca*. 200 clones per plate). Fusions conferring ampicillin resistance in these conditions were those made at the positions of the residues Gly42, His110, Gly176, Ala191, Glu194, Ser215, and Phe273, demonstrating that these BacA residues were located in a periplasmic environment (Table 3). The other fusions at residues Gly88, Lys140, Glu143, Pro144, Thr244, and Arg251 did not confer ampicillin resistance and were thus considered to have the β-lactamase, and these residues of BacA, in a cytoplasmic environment (Table 3). To exclude the possibility that some of the fusions failed to confer ampicillin resistance because no hybrid protein was made, rather than because the fusion junction was intracellular, the specific activity of β-lactamase was determined in membrane extracts prepared from these different transformants. While no activity was detected in cells carrying the empty pNF150 vector or the vector carrying the entire *bacA* gene alone, all the transformants expressing a BacA-BlaM protein exhibited a significant level of β-lactamase activity (Table 3), demonstrating that all these fusions were produced and functional. Furthermore, the capability of all of these plasmids to confer ampicillin resistance at a high cell density was tested. Indeed, as described previously [27], ampicillin-induced lysis of some cells releases the intracellular β-lactamase which could then hydrolyze the antibiotic locally and allow the neighboring cells to grow. We found that all the transformants were able to grow when patched on 2YT-ampicillin medium, confirming the expression of a functional hybrid protein in all cases.

**Table 3:** Construction and characterization of BacA-BlaM hybrid proteins. Truncated forms of the *bacA* gene were amplified by PCR by using the forward primer *bacA*-for and one of the reverse primers listed here whose sequences are reported in S1 Table. These fragments were inserted into the pNF150 vector and the resulting plasmids were transformed in the DH5α strain. The levels of C_55_-PP phosphatase and β-lactamase activities detected in membrane extracts of these transformants, as well as their susceptibility to ampicillin, were then determined.

Plasmid	3’end primer^a^	Fusion site^b^	Ampicillin resistance^c^	C_55_-PP phosphatase activity^d^	β-lactamase activity^e^
pNF150 vector	-	-	S	0.32	ND
pB42TEM	*bacA*-G42	Gly42	R	0.35	185
pB88TEM	*bacA*-G88	Gly88	S	0.39	203
pB110TEM	*bacA*-H110	His110	R	0.34	114
pB140TEM	*bacA*-K140	Lys140	S	0.36	321
pB143TEM	*bacA*-E143	Glu143	S	0.38	253
pB144TEM	*bacA*-P144	Pro144	S	0.31	715
pB176TEM	*bacA*-G176	Gly176	R	0.38	103
pB191TEM	*bacA*-A191	Ala191	R	0.39	32
pB194TEM	*bacA*-E194	Glu194	R	0.33	68
pB215TEM	*bacA*-S215	Ser215	R	0.30	43
pB244TEM	*bacA*-T244	Thr244	S	0.33	85
pB251TEM	*bacA*-R251	Arg251	S	0.39	126
pBacATEM	*bacA*-F273	Phe273	R	12.44	1563
pNF150::*bacA*	-	-	S	8.95	ND

^a^ The sequences of oligonucleotides are shown in S1 Table.

^b^ The fusion site corresponds to the C-terminal amino acid residue of the truncated BacA protein that has been fused to the mature form of β-lactamase via an intermediate peptide linker AVPHAISSSPLR originating from the pNF150 vector sequence.

^c^ DH5α strains carrying these different BacA-BlaM fusion-expressing plasmids were grown in 2YT- kanamycin liquid medium and dilutions (*ca*. 200 cells) were spread onto 2YT plates containing or not ampicillin at 100 μg.ml^-1^. Their resistance (R) or sensitivity (S) towards ampicillin was judged after 24 h of incubation at 37°C.

^d^ The total C_55_-PP phosphatase activity detected in membrane extracts of DH5α cells carrying these plasmids was determined as described in Materials and Methods and is expressed in nmol.min^-1^.mg^-1^ protein.

^e^ The β-lactamase activity detected in membrane extracts of DH5α cells carrying these plasmids was determined as described in Materials and Methods and is expressed in units.mg^−1^ protein. ND, not detected.

**Fig 5:**
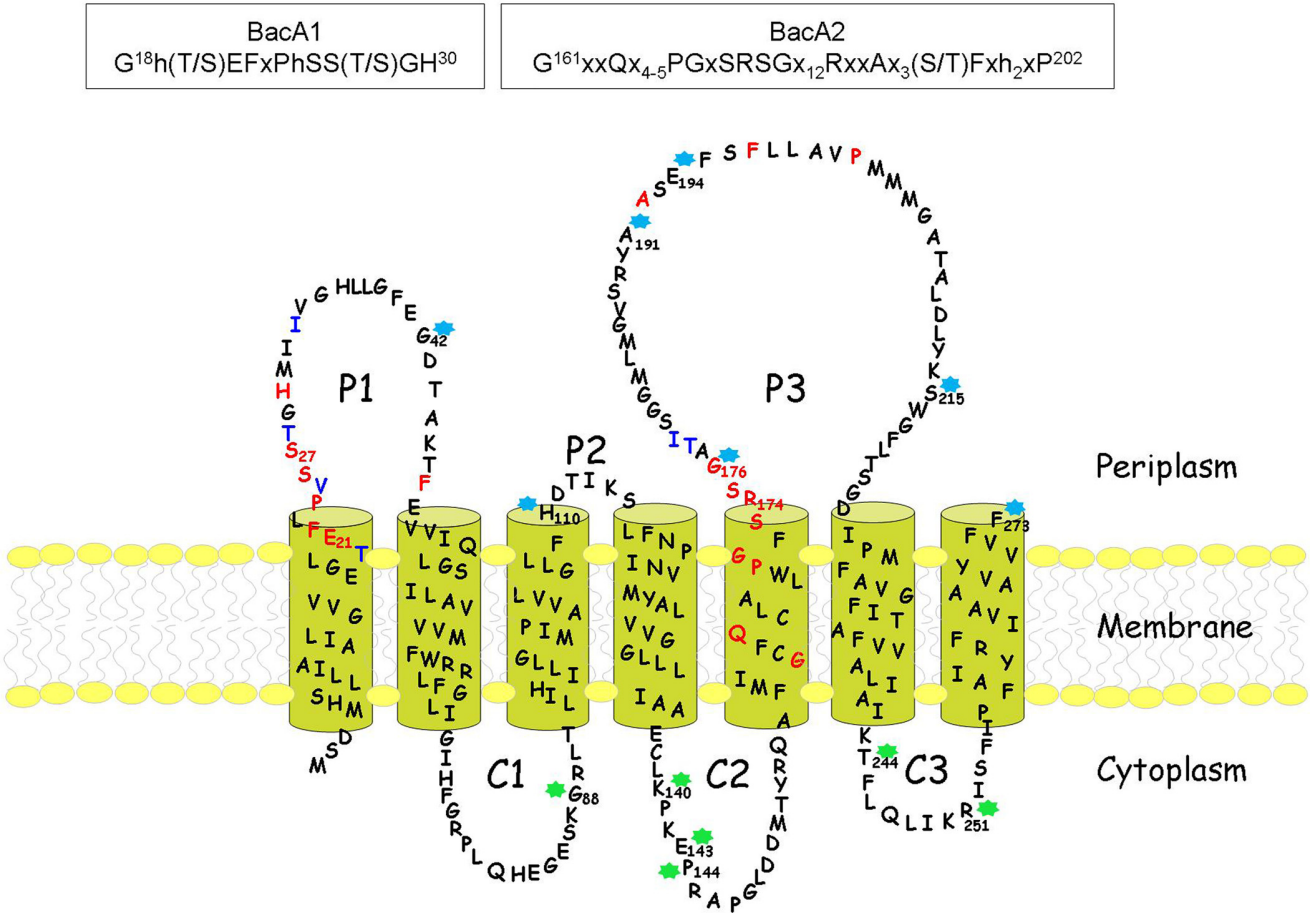
Membrane topology of the *E*. *coli* BacA protein. This topological model was determined experimentally as described in Materials and Methods by using the β-lactamase as a reporter of the transmembrane topology of BacA. P1-P3: periplasmic domains; C1-C3: cytoplasmic domains. Transmembrane segments were predicted by the TMAP program [25, 26]. BacA residues colored in red and blue are invariant and highly-conserved residues, respectively. Stars indicate the residues that were used as junction points for the construction of the different BacA-BlaM hybrid proteins. Blue and green stars correspond to hybrid proteins that conferred and did not confer ampicillin resistance to cells when plated at a low cell density, respectively. The sequences of the two characteristic BacA motifs (BacA1 and BacA2) are shown in the two boxes (invariant residues are in capitals, h correspond to hydrophobic residues and x to any residue; numbers in superscript indicate the position of these residues in the *E*. *coli* BacA protein sequence).

All these data generated experimentally allowed us to draw a topological model that diverged significantly from the previously predicted models [17, 18]. In particular, contrary to what had been proposed, it consisted of seven and not eight transmembrane segments and consequently the C-terminal extremity of the protein was in the periplasm and not in the cytoplasm (Fig 5). The fact that the fusion of the entire *bacA* gene to the β-lactamase gene in the pNF150 vector (pBacATEM plasmid) yielded an enzymatically-active hybrid protein, as judged by a *ca*. 30-fold increase of the C_55_-PP phosphatase activity detected in cell membranes, which conferred high-level ampicillin resistance to cells, clearly demonstrated that the C-terminal residue of BacA (Phe273) was localized in the periplasm (Table 3). As a confirmation, the latter plasmid was also shown to fully complement the growth defect of the thermosensitive BWTsbacA mutant strain. None of the other hybrid proteins, and in particular the fusion at Arg251 that only lacks the last 22 residues of BacA forming the seventh transmembrane segment, yielded such an increase of membrane C_55_-PP phosphatase activity nor complemented the thermosensitive mutant strain, showing that the whole protein sequence is required for the enzyme activity (Table 3). In the topological model thus generated, the highly-conserved BacA1 and BacA2 signature motifs were both located on the periplasmic side of the plasma membrane: the BacA1 motif was found at the end of the first transmembrane segment and in the P1 periplasmic loop, and the larger BacA2 motif was located within the fifth transmembrane segment and P3 periplasmic loop (Fig 5).

## Discussion

Based on their membrane topology, the PAP2 C_55_-PP phosphatases PgpB, YbjG and YeiU/LpxT were expected to participate exclusively in the recycling of C_55_-PP molecules that are released on the outer side of the membrane [14, 16]. It was thus speculated that the BacA protein, which belonged to a distinct protein family and lacked any feature of a typical phosphatase, should have a cytoplasm-orientated active site and be responsible for the *de novo* synthesis of the carrier lipid [1, 16]. In fact, the membrane topology of BacA determined here experimentally clearly shows that this enzyme also exerts its activity in the periplasm, raising the question of the identity of the enzyme catalyzing the dephosphorylation of the *de novo* synthesized C_55_-PP molecules (Fig 1). Different hypotheses can be formulated: (i) either another non-identified enzyme(s) specifically catalyzes this reaction on the cytoplasmic side of the membrane, or (ii) any one of the BacA, YbjG or PgpB proteins can perform it in the course of *de novo* synthesis as well as recycling. The latter hypothesis implies that newly made C_55_-PP molecules first have to be flipped towards the periplasm to be dephosphorylated and then are translocated back to the cytoplasm to be glycosylated. As shown here and previously [18], the BacA phosphatase could efficiently dephosphorylate C_15_-PP which is one of the two soluble substrates used by the UppS synthase to generate the C_55_-PP carrier lipid precursor. This side activity of BacA would have been deleterious for cells if the BacA active site was exposed towards the cytoplasm. Indeed, a significant part of the pool of C_15_-PP would be converted into a C_15_-P end product that do not exist normally in cells, resulting in an inhibition of UppS activity, a decreased availability of C_55_-P and loss of cell viability. This raises the question of the substrate specificity of the “missing” putative C_55_-PP phosphatase acting on the cytoplasmic side of the membrane, if such an enzyme exists, which must be highly specific to C_55_-PP to avoid such a toxic effect. In fact, this important point can make us envisage the C_55_-P biosynthesis/recycling process differently. As mentioned above and described in Fig 1, we can indeed speculate that the dephosphorylation of both the recycled and the *de novo*-synthesized C_55_-PP molecules exclusively occurs on the outer side of the membrane. This model does not involve any C_55_-PP phosphatase working intracellularly. It will instead need a flip-flop of the newly made C_55_-PP molecules before they can be provided in the form of C_55_-P for the synthesis of cell-wall polymers. What could be considered at a first glance as a futile energy-consuming step could in fact have several advantages. There is no need in that case for a cytoplasmic C_55_-PP phosphatase and consequently no problem of toxicity resulting from a broad-range substrate specificity of this enzyme, as mentioned above. That the C_55_-PP synthesis and dephosphorylation reactions occur in two distinct and specific cell compartments, *i*.*e*. on the inner and outer sides of the membrane, respectively, would confer a driving force for unidirectional transbilayer movement of the lipid, *i*.*e*., an accumulation of C_55_-PP in the inner leaflet would favor its translocation towards the outer leaflet, with the opposite way for C_55_-P. In any case, the identity of a C_55_-PP and/or C_55_-P flippase remains a central issue. It was speculated that the polyprenol-sugar flippase (FtsW, MurJ, *etc*.) [4, 5] could play this role but we can also envisage that the integral membrane C_55_-PP phosphatases themselves could actively participate in this process, with or without the aid of a flippase. The fact that the C_55_-PP dephosphorylation reaction occurs on only one membrane side would be more adapted for the development of potential regulatory mechanisms adjusting the C_55_-P pool to the specific requirements of the biosynthesis of the different cell-wall polymers.

**Fig 6:**
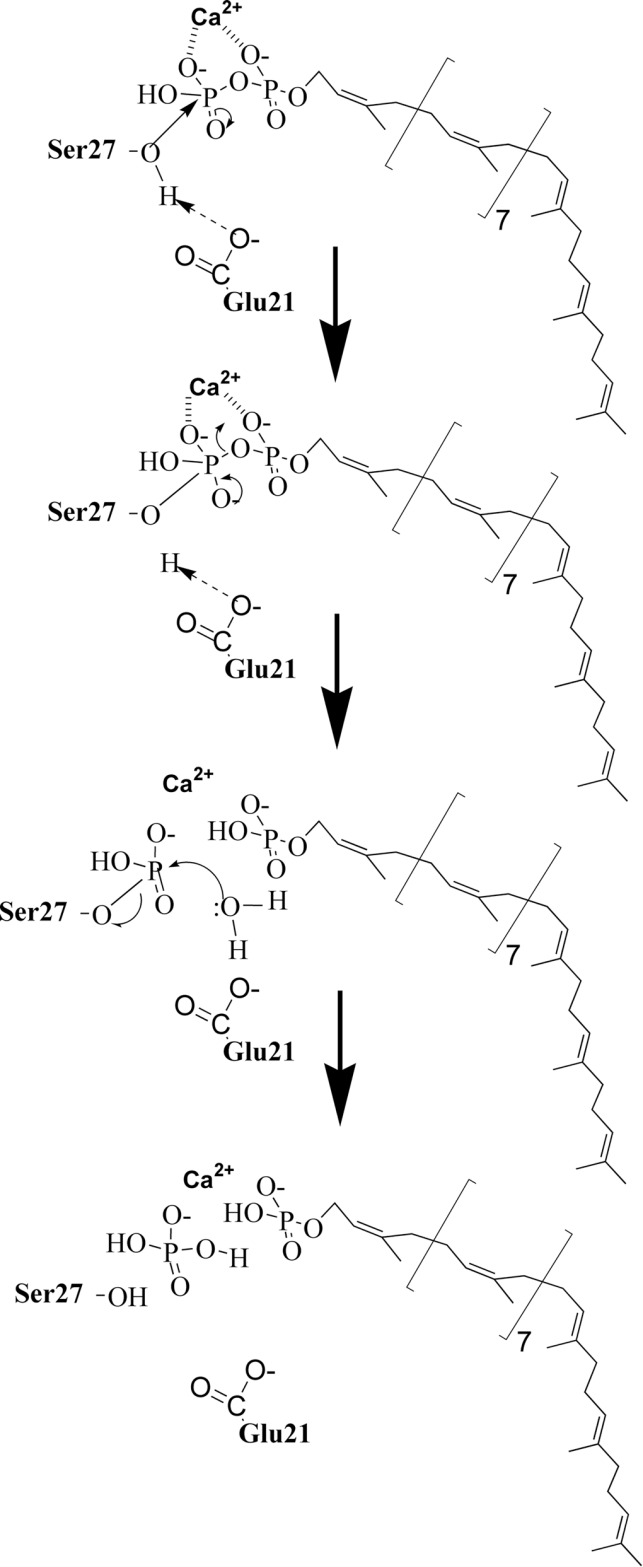
Proposed catalytic mechanism for the BacA C_55_-PP phosphatase. The present mechanism is based on the results of the structure-activity relationship analysis developed in this work. Two residues Glu21 and Ser27 that are essential for the catalytic process were identified, whose mutation into alanine yielded BacA enzymes with dramatically altered activities (1.4% and 0.013% of residual activity, respectively). The Ser27 residue has a hydroxyl group that can act as a nucleophile when deprotonated by the carboxyl group of Glu21 residue, the latter group displaying a negative charge at the pH that is optimal for BacA activity. The deprotonated Ser27 then initiates a nucleophilic attack on the β-phosphorus center of C_55_-PP substrate to form a phospho-serine enzyme intermediate, a reaction releasing the C_55_-P product. A subsequent attack by a water molecule releases the second inorganic phosphate product.

In contrast to the PAP2 C_55_-PP phosphatases, BacA enzymes do not belong to a family of characterized enzymes, neither structurally nor mechanistically. Sequence alignment of a great number of bacterial BacA homologues revealed two regions of the protein displaying a few invariant amino acid residues (Fig 4). In order to identify the catalytic site residues and to figure out the possible catalytic mechanism of BacA, we as well as Chang *et al*. [18] performed a structure-activity relationship analysis by using a site-directed mutagenesis approach. Discrepancies were observed between these two studies which may be explained by the fact that, to test the enzymatic activity of their mutated proteins *in vitro*, Chang *et al*. used C_15_-PP as a substrate, *i*.*e*. the soluble C_55_-PP precursor. In contrast, we used the natural substrate C_55_-PP that we synthesized by using the purified *E*. *coli* UppS enzyme. It is reasonable to figure out that the polyprenol chain itself may contribute in the recognition of the lipid by the membrane-embedded phosphatase. Moreover, we experienced, with the PgpB enzyme, that the kinetics of hydrolysis of C_5_-PP and C_55_-PP *in vitro* were strikingly different [14]. Whereas the velocity of hydrolysis of C_55_-PP was decreasing as the amount of detergent was raising, that of C_15_-PP did not vary with the detergent concentration. The C_55_-PP hydrolysis is referred as surface dilution kinetic that likely reflects the dilution of the lipid substrate at the surface of the micelles [31], while C_15_-PP, despite its 15-carbon chain length aliphatic chain, likely remains in a soluble state in the aqueous environment. As a consequence, the turnover of hydrolysis of C_15_-PP was only dependent on its bulk concentration. Therefore, the mode of access of the lipid substrates to the active site of the phosphatases in the detergent-micelle model must be extremely different, with that of C_55_-PP likely being more physiologic than that of C_15_-PP. Our BacA mutagenesis study was strengthened by the fact that the results we obtained *in vitro* could be substantiated by a complementary *in vivo* analysis. Indeed, the functionality of our variants was tested via a complementation assay by using our conditional mutant strain BWTsbacA inactivated for all three C_55_-PP phosphatase-encoding genes and expressing *bacA* on a temperature-sensitive plasmid. We then demonstrated that the substitution of Glu21, Ser27 and Arg174 residues by an alanine almost completely inactivated the enzyme both *in vivo* and *in vitro*, without causing major structural instability. The substitutions of two other residues, His30 and Ser173, partially inactivated the enzyme (5–10% residual activity). In contrast, Chang *et al*. found that the mutations of His30, Ser173, Arg174 and Thr178 totally inactivated the enzyme when tested *in vitro* towards C_15_-PP [18]. This finding led them to hypothesize that His30 residue may be a catalytic residue acting as the nucleophile in the attack on the phosphorus center to form a phospho-histidine intermediate [18]. In fact, we here show that the His30 residue is not at all essential for catalysis, contrary to what would be expected for such a mechanism. Noteworthy, in contrast to the PAP2 enzymes, BacA phosphatase activity appeared to be dependent upon the presence of a cation as the enzyme was totally inhibited by EDTA [18]. We also observed that Ca^2+^ was by far the most efficient cation in supporting BacA activity (about 7-fold higher activity in presence of Ca^2+^ as compared to Mg^2+^, and almost no detectable activity with other divalent cations). Such a metal ion requirement has been observed for the C_55_-PP synthase, UppS enzyme, where Mg^2+^ was found to be essential for the catalysis by facilitating the nucleophilic attack of the C_15_-PP allylic substrate by C_5_-PP and the release of its pyrophosphate group [32]. The metal ion maintains the pyrophosphate moiety of the substrate in an optimal conformation for the enzyme catalysis. Based on the very strong decrease of BacA activity (0.013% of residual activity) that is observed following mutagenesis of the Ser27 residue, we suggest that this essential serine should act as a nucleophile residue in the attack of the C_55_-PP β-phosphorus atom, thereby yielding a phospho-seryl intermediate and releasing C_55_-P. The charged carboxyl group of the side chain of Glu21 residue may ensure the deprotonation of the hydroxyl group of the catalytic Ser27 residue to form a reactive oxyanion. This specific role proposed for Glu21 is supported by the enzymatic activity of the E21A mutant protein and by the optimal pH (5–7.5) of the BacA-catalyzed reaction which is compatible with the p*K*_a_ of its charged group (4.07). Subsequently, the phospho-enzyme must be hydrolyzed through the action of an activated water molecule or another nucleophile, thus releasing inorganic phosphate or a phosphorylated acceptor molecule, respectively (Fig 6). The positively charged guanidino group of the Arg174 residue could be important in holding the pyrophosphate group of the lipid substrate. Future work and in particular attempts to trap such a covalently-bound phospho-enzyme intermediate, by using the different mutants and/or substrate analogues, will provide valuable insights into the catalytic mechanism of this enzyme. Of course the elucidation of the three-dimensional structure of the BacA protein will constitute a major challenge and milestone, and the acquired knowledge on the purification, biochemical properties and stability of this protein will likely be very helpful in this regard.

## Supporting Information

S1 Table Oligonucleotides used in this study. (PDF)
(DOC)
