# Supplementary material for: Membrane Topology and Biochemical Characterization of the Escherichia coli BacA Undecaprenyl-Pyrophosphate Phosphatase
Source: PLoS One. 2015 Nov 11;10(11):e0142870. doi: 10.1371/journal.pone.0142870 (PMC4641660; doi:10.1371/journal.pone.0142870)
Supplement: S1 Table — (PDF) (DOC) [file pone.0142870.s001.doc]

Supporting Information

**S1 Table. Oligonucleotides used in this study.**

| Oligonucleotides | 5’ to 3’ Sequence^a^ |
| --- | --- |
| ***topology***  *bacA*-for  *bacA*-G42  *bacA*-G88  *bacA*-H110  *bacA*-K140  *bacA*-E143  *bacA*-P144  *bacA-*G176  *bacA*-A191  *bacA*-E194  *bacA*-S215  *bacA*-T244  *bacA*-R251  *bacA*-F273  ***mutagenesis***  E21Afor  E21Arev  S26Afor  S26Arev  S27Afor  S27Arev  H30Afor  H30Arev  Q164Afor  Q164Arev  S173Afor  S173Arev  R174Afor  R174Arev  S175Afor  S175Arev  R189Afor  R189Arev  S196Afor  S196Arev | TTAAGGATCCAGGAgTTTATTgATGagcgatatgcactcg  TTTCGGTACCGCGCCCTCAAACCCCAACAAGTGACC  CAGCGGTACCGCACCTTTGCTTTCACCTTCGTGCTG  CTTAGGTACCGCGTGGAACAACAGCCCCAATACCACC  CGGCGGTACCGCCTTCAGGCATTCGGCGGCAATCAG  CGGCGGTACCGCCTCTTTCGGCTTCAGGCATTCGGC  ACCCGGTACCGCCGGCTCTTTCGGCTTCAGGC  TGAAGGTACCGCCCCGGAACGGGAGAAACCCGG  AAACGGTACCGCAGCGTAACGGCTCACCCCCATC  CAGGGGTACCGCCTCGGAAGCAGCGTAACGGCTCACC  CAGGGGTACCGCGCTTTTGTAGAGATCGAGCGC  CAATGGTACCGCGGTTTTAATCGCTATCAGCGC  GATAGGTACCGCGCGCTTAATCAATTGCAGGAAGG  ATTCGGTACCGCAAAGAACACGACATACACCGC  GGTGTGGTCGAAGGATTGACA**GCA**TTTCTGCCGGTATCCAGC  GCTGGATACCGGCAGAAA**TGC**TGTCAATCCTTCGACCACACC  GAATTTCTGCCGGTA**GCC**AGCACGGGCCATATG  CATATGGCCCGTGCT**GGC**TACCGGCAGAAATTC  GAATTTCTGCCGGTATCC**GCC**ACGGGCCATATGATTATTG  CAATAATCATATGGCCCGT**GGC**GGATACCGGCAGAAATTC  CCGGTATCCAGCACGGGC**GCT**ATGATTATTGTCGG  CCGACAATAATCAT**AGC**GCCCGTGCTGGATACCGG  GCATTTATGATTGGCTGTTTC**GCG**TGTCTGGCGCTGTGG  CCACAGCGCCAGACA**CGC**GAAACAGCCAATCATAAATGC  GCTGTGGCCGGGTTTC**GCC**CGTTCCGGGGCGACC  GGTCGCCCCGGAACG**GGC**GAAACCCGGCCACAGC  CTGTGGCCGGGTTTCTCC**GCT**TCCGGGGCGACCATTTCAGG  CCTGAAATGGTCGCCCCGGA**AGC**GGAGAAACCCGGCCACAG  GCGCTGTGGCCGGGTTTCTCCCGT**GCC**GGGGCGACCATTTCAGGTGG  CCACCTGAAATGGTCGCCCC**GGC**ACGGGAGAAACCCGGCCACAGCGC  GCTGATGGGGGTGAGC**GCT**TACGCTGCTTCCGAG  CTCGGAAGCAGCGTA**AGC**GCTCACCCCCATCAGC  TACGCTGCTTCCGAGTTT**GCG**TTCCTGCTGGCGGTGCCG  CGGCACCGCCAGCAGGAA**CGC**AAACTCGGAAGCAGCGTA |

^a^Restrictions sites for BamHI (GGATCC) and KpnI (GGTACC) that were introduced in oligonucleotide sequences are underlined and codons that were mutagenized in the *bacA* gene sequence are in bold.
